# Peptide Therapeutics 2.0

**DOI:** 10.3390/molecules25102293

**Published:** 2020-05-13

**Authors:** Beatriz G. de la Torre, Fernando Albericio

**Affiliations:** 1 KwaZulu-Natal Research Innovation and Sequencing Platform (KRISP), School of Laboratory Medicine and Medical Sciences, College of Health Sciences, University of KwaZulu-Natal, Durban 4041, South Africa; 2 Peptide Science Laboratory, School of Chemistry and Physics, University of KwaZulu-Natal, Westville, Durban 4000, South Africa; 3 CIBER-BBN, Networking Centre on Bioengineering, Biomaterials and Nanomedicine, Institute for Advanced Chemistry of Catalonia (IQAC-CSIC), 08034 Barcelona, Spain; 4 Department of Organic Chemistry, University of Barcelona, Martí i Franqués 1-11, 08028 Barcelona, Spain

In recent years, the peptide drug discovery field has shown a high level of dynamism, with hundreds of academic groups working on this topic, the creation of new peptide-focused companies, and the consolidation of peptide business by so-called big pharma [1,2,3,4].

In the last five years (2015–2019), the U.S. Food Drug Administration (FDA) have authorized a total of 208 new drugs (150 new chemical entities and 58 biologics) [5,6], 15 of which were peptides or peptide-containing molecules (Table 1), which account for 7% of the total number of drugs [4,7]. This is a rather impressive number, if we consider the efforts of the pharmaceutical industry in peptides in comparison to small molecules (in the context of this work, a peptide is defined as a compound that contains two or more amino acids linked by an amide (peptide) bond and that can be synthesized chemically).

The chemical structure and medical indication of the active principle ingredient of these drugs show an excellent representation of the diversity of the peptide world.

From a chemical structure perspective, it is possible to find small peptides (Ninlar^®^, Macrilen^®^); medium-sized peptides (Giapreza^®^, Scenesse^®^); homodetic (through amide bonds) cyclic peptides (Vyleesi^®^); intra- and intermolecular disulfide-containing peptides (Parsabiv^®^, containing almost exclusively D-amino acids; Trulance^®^); large peptides (Tymlos^®^, Lixisenatide^®^), which in some cases are branched (Ozempic^®^, Tresiba^®^); and peptides containing radionuclides [Lutathera^®^, 68Ga DOTA-TOC (^68^Ga-labeled 1,4,7,10-tetraazacyclododecane-N,N’,N’’,N’’’-tetraacetic acid-D-Phe1-Tyr3-octreotide)]. In the case of the two antibody drug conjugates (ADC) PADCEV^®^ and Polivy^®^, the payload is the peptide monomethyl auristatin E (MMAE), a synthetic analog of the marine natural peptide dolastatin 10. MMAE is also the drug contained in Adcetris^®^, which was approved by the FDA in 2011. Of the seven FDA-approved ADCs to date, three contain a peptide. Moreover, PADCEV^®^ and Polivy^®^ contain the dipeptide Val-Cit as a linker. Another peptide-based linker, Gly-Gly-Phe-Gly, is present in the ADC Enhertu^®^, which was authorized by the FDA in 2019.

Oncology, with five drugs (two radio peptides and two ADCs), metabolism (three), and endocrinology (two) are the most frequent medical indications for peptides. However, cardiovascular conditions, gastroenterology, bone diseases, dermatology, and sexual dysfunction are also targeted by peptides.

Of note, between 2015 and 2019, several of the new peptide-based drugs accepted by the FDA came about from the efforts of academic groups. This highlights the importance of fostering solid and efficient cooperation channels between academia and industry with the aim to maintain and improve the well-being of society.

In addition to the use of peptides as drugs or in diagnostics, these molecules are playing an increasingly important role as drug delivery systems and as the base for new biomaterials with broad potential applications in medicine.

This analysis supports the strength of peptides in the medicinal field. In this context, we have decided to publish a Special Issue in Molecules, termed “Peptide Therapeutics 2.0”, which contains excellent quality research articles and comprehensive reviews on peptides. It is hoped that some of the peptides introduced herein will reach the market in the coming years.

## Figures and Tables

**Table 1:** Peptide-based drugs approved by the Food Drug Administration (FDA) (2015–2019) [3,4,5,6].

Year	Active Ingredient Trade Name	Indication	Features
2015	Insulin degludec Tresiba^®^	Diabetes	Modified insulin with an aa deletion and a hexadecanedioic acid via γ-Glu at the Lys (B29)
2015	Ixazomib Ninlar^®^	Multiple myeloma	*N-*Acylated, *C-*boronic acid dipeptide
2016	Adlyxin Lixisenatide^®^	Diabetes	44 aa GLP-1 peptide with (Lys)_6_ at the *C*-terminal
2017	Abaloparatide Tymlos^®^	Osteoporosis	34 aa analog of parathyroid hormone-related protein
2017	Angiotensin II Giapreza^®^	Hypotension	Natural octapeptide
2017	Etelcalcetide Parsabiv^®^	Hyperparathyroidism	Ac-DCys-DAla-(DArg)_3_-DAla-DArg-NH_2_ linked to L-Cys through a disulfide bridge
2017	Macimorelin Macrilen^®^	Growth hormone deficiency	Pseudotripeptide *N-*formylated
2017	Plecanatide Trulance^®^	Chronic idiopathic constipation	16 aa with two disulfides
2017	Semaglutide Ozempic^®^	Diabetes	GLP-1 peptide (31 aa in the chain) with hexadecanedioic acid via γ-Glu and mini PEG at Lys
2018	177Lu DOTA-TATE Lutathera^®^	Neuroendocrine tumors, theranostic	177Lu chelated by DOTA bound to Tyr3-octreotate
2019	68Ga DOTA-TOC	Neuroendocrine tumors, diagnostic	68Ga chelated by DOTA bound to Tyr3-octreotide
2019	Afamelanotide Scenesse^®^	Skin damage and pain	13 aa lineal peptide analog of α-MSH
2019	Bremelanotide Vyleesi^®^	Women hypoactive sexual desire	7 aa cyclic peptide analog of α-MSH
2019	Enfortumab Vedotin-Ejfv PADCEV^®^	Cancers expressing Nectin-4	ADC with a synthetic analog of the marine natural peptide dolastatin 10
2019	Polatuzumab Vedotin-Piiq Polivy^®^	Diffuse large B-cell lymphoma	ADC with a synthetic analog of dolastatin 10 (5-residue peptide alcohol)
